# Somatosensory evoked fields at 36 weeks' gestation in preterm infants: A pilot study of thalamocortical maturation

**DOI:** 10.1016/j.cnp.2026.08.003

**Published:** 2026-08-11

**Authors:** Stephanie C. Schüssler, Fabian B. Fahlbusch, Undine Niederreiter, Martin Kaltenhäuser, Heiko Reutter, Regina Trollmann, Stefan Rampp

**Affiliations:** a Department of Pediatrics and Adolescent Medicine, Pediatric Neurology, Friedrich-Alexander-Universität Erlangen-Nürnberg, Germany; b Neonatology and Pediatric Intensive Care, Medical Faculty, University of Augsburg, Augsburg, Germany; c Department of Neurosurgery, Friedrich-Alexander-Universität Erlangen-, Nürnberg, Germany; d Department of Pediatrics and Adolescent Medicine, Neonatology and Pediatric intensive care, Friedrich-Alexander-Universität Erlangen-, Nürnberg, Germany

**Keywords:** Preterm infants, Magnetoencephalography, Somatosensory evoked fields (SEF), Neurodevelopmental maturation, White matter development

## Abstract

**Objective:**

Somatosensory evoked fields (SEF) recorded using magnetoencephalography enable non-invasive measurement of the cortical activity within the afferent somatosensory pathway. Because MRI studies suggest that delayed myelination and damage to the white matter contribute to adverse effects on neurological development in preterm infants, early functional markers of brain maturation are of particular interest.

**Methods:**

In this single-center pilot study, SEFs were recorded using tactile stimulation in 13 preterm infants examined at 36 weeks' gestational age (GA) and 8 term infants examined at 40 weeks' GA. Latencies to the first cortical deflection in the somatosensory cortex were analyzed and compared across gestational ages at recording and between very preterm (26–31 weeks of GA at birth) and moderate/late preterm infants (32–36 weeks GA).

**Results:**

SEF waveforms were obtained in all infants. Latencies were longer in preterm infants at 36 weeks' GA compared to term infants at 40 weeks' GA and decreased significantly with advancing GA. No significant latency differences were found between very preterm and moderate/late preterm infants at 36 weeks' GA.

**Conclusions:**

MEG-based SEF recording is feasible in preterm infants at 36 weeks' GA. SEF latency did not provide evidence of a significant delay in somatosensory maturation in very preterm compared with moderate/late preterm infants and does not support the hypothesis of a delayed myelination in very preterm infants.

**Significance:**

Although limited by sample size, single time-point measurements, and lack of magnetic resonance imaging (MRI), our study lays a foundation for future MEG investigations of premature infants in early life.

## Highlights

• First study on somatosensory evoked fields latencies at 36 weeks´ gestational age (GA).
• Provides functional data on somatosensory pathway development and myelination.
• Similar latencies in very preterm and late preterm infants at 36 weeks´ GA.

## 1 Introduction

Despite major advances in perinatal and neonatal care – including antenatal corticosteroids, surfactant administration, modern respiratory support, and improved nutritional strategies - neurodevelopmental impairments remain common in survivors of extreme prematurity (Stoll et al., 2015; Molloy et al., 2024; Saigal and Doyle, 2008). These sequelae range from learning difficulties and subtle motor problems to severe cerebral palsy and intellectual disability (Pierrat et al., 2021; Serenius et al., 2016). Recent epidemiological studies have shown that 41–47% of infants born at 24–29 weeks' gestation exhibit intellectual deficits, and infants born before 32 weeks face a 5.3–8.8% risk of cerebral palsy and an 8.6–10.1% risk of motor impairment (Pierrat et al., 2021; Hafstrom et al., 2018). While cerebral palsy is strongly linked to intracerebral haemorrhage and periventricular leukomalacia (Rees et al., 2022), the mechanisms underlying later cognitive impairment are far less clear.

### 1.1 White matter injury (WMI) and the hypothesis of delayed myelination

Altered myelination of the cerebral white matter is considered a key candidate mechanism for neurocognitive difficulties in former preterm infants. White matter injury is detectable on MRI in up to one third of infants born between 24 and 32 weeks and is associated with a 3.48-fold higher risk of cognitive impairment (Back, 2017; Rees et al., 2022). Within the CNS, myelination is driven by oligodendrocytes whose precursor cells are exquisitely vulnerable to oxidative stress, in contrast to relatively resilient immature neurons (Back, 2017). Myelination depends on an optimal metabolic and oxygen milieu (Grotheer et al., 2023). Although MRI remains the gold standard for assessing myelination, differences across techniques—including fractional anisotropy and myelin water fraction imaging—limit comparability (Soun et al., 2017). A recent MRI study using T1w/T2w ratios reported significantly reduced myelination after preterm birth and hypothesised that extrauterine maturation occurs more slowly than intrauterine maturation (Grotheer et al., 2023).

### 1.2 Thalamocortical development and cognition in preterm infants

Beyond white matter injury, grey matter alterations—particularly in the thalamus—are suspected to contribute to cognitive impairment. The few studies that have tested this hypothesis using MRI tractography and functional connectivity analyses suggest disturbances in thalamocortical organisation in preterm infants (Ball et al., 2013). Ball et al. hypothesised that disrupted thalamocortical development may contribute to the cognitive phenotype of prematurity (Ball et al., 2013).

### 1.3 Somatosensory evoked potentials (SEP) and somatosensory evoked fields (SEF)

Somatosensory evoked potentials (SEP) provide information about the integrity and myelination of ascending sensory pathways—from the peripheral nerve through the thalamus to the primary somatosensory cortex. SEP recordings in preterm infants were first described as early as 1973 (Hrbek et al., 1973). In adults, cortical responses are characterized by the N20 component at ∼20 ms, whereas newborns show a slower negative deflection (Hrbek et al., 1973; Trollmann et al., 2010).

In SEP, voltage differences are recorded with EEG electrodes. In contrast, Magnetoencephalography captures minute magnetic fields generated by intracellular currents in cortical neurons, providing excellent temporal and spatial resolution (Burgess et al., 2011). Due to the high sensitivity of the MEG sensors, a tactile stimulus is sufficient to obtain a measurable cortical response in the somatosensory cortex (Lauronen et al., 2006), which in newborns typically occurs around 60 ms (Pihko et al., 2009). Nevalainen et al. first demonstrated that tactile stimulation elicits robust somatosensory evoked fields (SEF) in preterm and term infants (Nevalainen et al., 2008). In contrast, it was not possible to obtain a reliable N1 response to tactile stimulation in the EEG of healthy newborns, probably due to a reduced sensitivity of the method (Pihko et al., 2004). Therefore, in SEP, it is standard practice to stimulate peripheral nerves electrically which can be unpleasant for neonates. The ability to measure brain responses using tactile stimulation is a major advantage for the vulnerable patient group of premature babies for vulnerable premature babies.

Prior MEG work compared SEF latencies at term age (∼40 weeks' GA), and reported no latency differences between formerly preterm and term-born infants (Nevalainen et al., 2008).

In contrast, conventional SEP studies found longer latencies associated with prolonged ventilation in premature infants (Tombini et al., 2009). Between the 37th and 41st week of pregnancy, the rapid physiological maturation of the somatosensory pathways is reflected in a shortening of the latency period (Trollmann et al., 2010). In particular, a difference in central latency from the cervical spinal cord to the cortex was observed. This amounted to 16.6 ms. The peripheral latency times for the median nerve, measured from the wrist to the recording site in the neck area, showed only a difference of one millisecond between premature and full-term babies. This finding suggests that differences in total latency are mainly due to differences in the central conduction time defined as the latency between cervical and cortical recording.

### 1.4 Relationship between SEF/SEP and cognitive development in preterm infants

The mechanisms leading to cognitive impairments in preterm infants remain unclear and are likely to be complex. Research is currently focused on identifying differences in brain development between preterm and term infants by MRI and functional imaging techniques such as fMRI, EEG, and evoked potentials (Grotheer et al., 2023, Ball et al., 2013, Kong et al., 2018). In particular, early somatosensory evoked responses have been investigated as functional markers of sensory pathway maturation and, in some studies, as predictors of later developmental outcome. A study including 86 preterm infants with an average gestational age of 27.9 weeks demonstrated that a normal SEP response at term was associated with a favourable cognitive outcome, assessed by the Bayley Scales of Infant and Toddler Development Version III (BSID-III) at the age of two years (Coviello et al., 2024). The prognostic value of SEP/SEF is limited, of course, as it provides information on the maturation of the afferent somatosensory system but does not directly reflect complex brain networks or whole-brain myelination.

### 1.5 Aim of the study

Existing SEF studies have only examined infants at ∼40 weeks' gestation, leaving earlier developmental windows unexplored. In our study we aimed to determine whether MEG-based SEF latency recordings at 36 weeks' GA are feasible in clinically stable preterm infants at 36 weeks' GA and whether SEF latencies differ between very preterm infants (postnatal age 5–8 weeks) and moderate/late preterm infants (postnatal age 1–4 weeks) at this age. Our hypothesis was that very preterm infants would show prolonged SEF latencies compared to moderate/late preterm infants, reflecting reduced intrauterine maturation time and potentially delayed myelination or altered maturation of the somatosensory pathway.

## 2 Methods

### 2.1 Study setting and participants

This study was conducted at the Department of Pediatrics and Adolescent Medicine, Friedrich-Alexander-University Erlangen-Nürnberg (FAU), Germany, a tertiary care center with specialized neonatology and pediatric neurology departments. Premature infants were recruited from the neonatal ward. Inclusion criteria were preterm birth and clinical stability allowing transport to the MEG facility on campus. Clinical data, including gestational age, birth weight, and details of neonatal care, were extracted from the electronic patient record system (Soarian, Siemens). The study group included term infants born with a gestational age of 37–39 weeks of GA, extreme preterm infants who were born before 32 weeks of GA and late preterm infants who were born after 32 weeks of GA. The measurement of the preterm infants was conducted when they had reached a gestational age of at least 35 weeks of GA.

### 2.2 Ethical approval and consent

The study was approved by the local ethics committee of the Friedrich-Alexander-University Erlangen-Nürnberg (ID number: 21-116_1B). Written informed consent for study participation was obtained from all parents prior to inclusion, in accordance with the Declaration of Helsinki.

### 2.3 Patient transport and preparation

For MEG recording, infants were transported from the NICU to the magnetoencephalography laboratory in a stroller. The MEG system was not specifically designed for neonatal recordings, but was an adult whole-head MEG system equipped with a patient table. Therefore, we placed a custom wooden support board between the patient bed and the Dewar on the MEG to allow positioning of the neonate directly beneath the sensor array. A photograph of the recording setup is provided in the e-supplement. Infants were wrapped in a warm blanket to make them feel warm and cozy. All infants wore soft baby hats. To protect the skins of the neonates, the fiducial points were drawn on small adhesive bandages and stuck onto the children's hats: two of them pre-auricular, one in the middle of the forehead extending upwards of the nasion, one in a central position and one in occipital position. In the Cartesian coordinate system, the y-axis passed through the pre- auricular points with the positive y-direction pointing to the left; the x-axis passed through the nasion with the positive x-direction pointing to the nasion; thus the positive z-direction was upwards.

### 2.4 MEG recording and somatosensory stimulation

MEG recordings were obtained using a 248-magnetometer sensor whole-head system (Magnes 3600 WHS; 4D-Neuroimaging, San Diego, CA, USA) in a magnetically and electrically shielded room (Vacuumschmelze, Hanau, Germany). Using a digitization pen (Polhemus, Colchester, VT, USA), five landmark points were marked to setup a subject coordinate system: left and right preauricular point, nasion, inion and vertex. Further 50–100 points (“headshape”) were marked on the forehead to support coregistration with MRI templates. The infants were placed in a lateral position for measurement, with one side of their head resting on the sensor head. When we stimulated the right hand, the left side of the head rested on the sensor; to measure the signal evoked at the left hand, we turned the child so that the right side of the head was in direct contact with the sensor head. The infants were asleep during the measurement or lay quietly just before falling asleep. We did not administer sedatives. Somatosensory stimulation was delivered pneumatically via a small balloon placed on the infant's fingers, connected to the stimulator unit of the MEG system. The stimulator pressure reaches 1.155 bar within 10 ms. The number of stimuli per trial was 500 with an interstimulus interval (ISI) of 500 ms. Cerebral evoked magnetic fields were sampled in epochs 500 ms before and 500 ms after the stimulus trigger with an analogue filter of 1-200 Hz. The sampling rate was set at 508.63 Hz.

### 2.5 Signal processing and SEF identification

Cardiac and blink artifacts were removed using Signal-Space-Projection (SSP) based on manual marking of artifacts (Tesche et al., 1995; Uusitalo and Ilmoniemi, 1997). The median number of rejected trials per infant was 40 (1./3. quartile 25–97, minimum 0, maximum 223). However, even in the case in which the maximum number of trials were rejected, the remaining are well within the recommended range of 200–500 trials (Burgess et al., 2011). Continuous data was segmented into epochs, ranging from 500 ms before to 500 ms after the stimulus. Baseline correction was performed by calculating the mean baseline shift of the pretrigger (<0 ms) interval per channel and subtraction from the complete epoch. Subsequently, epochs were low-pass and notch-filtered at 70 Hz, respectively 50 Hz. The comparably long pre- and post-stimulus segments ensured that edge effects of filtering did not overlap with the signal of interest. Channels and epochs containing excessive noise were excluded based on visual inspection. The remaining epochs were then averaged. To leverage higher signal-to-noise ratios, source imaging was performed at each timepoint using a single-dipole scan in a spherical head model and a template MRI based on coregistered averaged MRIs of 2-week-old newborns (O'Reilly et al., 2021). Coregistration with the MEG data was based on the digitized landmarks and headshape points. Power of source amplitudes over time within the postcentral parcel from the Desikan-Killiany atlas (Desikan et al., 2006) was determined by taking the average of the square of all signals contained in this parcel. The resulting time series were then inspected visually. Since there is no automatic detection for children of this age and the latencies, the peaks had to be identified visually and manually by the researchers. The peaks were identified by the author SR who is an experienced neurophysiologist and MEG expert researcher. The identified peaks were confirmed by SS who is an experienced child neurologist and clinical neurophysiologist. Data analysis was performed with Brainstorm (Tadel et al., 2011),which is documented and freely available for download online under the GNU general public license (http://neuroimage.usc.edu/brainstorm).

### 2.6 Neurodevelopmental follow-up

All preterm infants with a birth weight < 1500 g participated in the center's standardized neurological follow-up program, which was independent of the MEG study procedures. Assessments included expert neurological examinations and developmental testing using the Griffiths Developmental Scales (Griffiths, 1996; Huntley, 1996) or the cognitive composite scale of the BSID-III (Reuner, 2014) at the age of two years.

### 2.7 Statistical analysis

Statistical analyses were performed using SPSS Version 21 (IBM, Armonk, NY, USA) and GraphPad Prism Version 9 (GraphPad Software, Boston, MA, USA). The equality of variances was tested by the Levene's test. Group comparisons were performed using the One-way ANOVA and Dunn's multiple comparison post-hoc test. Associations between SEF latencies and clinical parameters (e.g., birth weight) were examined using linear regression analysis**.** *P* Values <0.05 were considered statistically significant.

## 3 Results

### 3.1 Cohort characteristics

A total of 21 infants were included (Table 1 and Table 2). The cohort consisted of 13 preterm infants, of whom 7 were very preterm (26–31 weeks' gestational age) and 6 were moderate to late preterm (32–36 weeks' GA). All preterm infants underwent MEG recordings at a postmenstrual age of 36 weeks. Additionally, 8 term infants were examined at 40 weeks' GA.

**Table 1:** Patient characteristics of preterm infants.

	Very preterm <32 SSW *N* = 7	Late preterm 32-37SSW *N* = 6	All preterm
GA at birth (weeks), mean (range)	29 + 0 (26 + 6–31 + 3)	33 + 5 (32 + 4–36 + 3)	31 + 3 (26 + 6–36 + 3)
Birth weight (g), mean (SD)	1111 ± 201	2100 ± 389	1568 ± 587
Head circumference at birth(cm), mean (SD)	26.6 ± 1.6	31.1 ± 1	28.7 ± 2.7
Age postnatal days, mean (range)	49 (36–62)	16 (5–27)	34 (6–62)
GA age at measurement, mean (range)	36 + 1 (35 + 4–36 + 6)	35 + 6 (35 + 2–37 + 1)	36 + 1 (35 + 2–37 + 1)
Duration of respiratory support (days), mean (range)	20.7 (11–47)	3.7 (0–9)	12.9 (0–47)
Intraventricular haemorrhage (n)^1^	2	0	2
36 week SEF Latency, ms	**102.5** **±** **6.3**	**97.3** **±** **17.1**	**100.1** **±** **12.2**

GA: gestational age, SD: standard deviation, ms: miliseconds. The values are given as mean values with standard deviation (SD) or range.

1 One infant born at 26 + 2 weeks' gestation had unilateral grade II IVH, and one infant born at 28 + 6 weeks' gestation had grade III IVH on the right and grade II IVH on the left.

**Table 2:** Patient characteristics of term infants.

	Term infants *N* = 8
GA at birth (weeks), mean (range)	38 + 5 (37 + 0–39 + 6)
Birth weight (g), mean (SD)	3256 ± 365
Head circumference at birth (cm), mean (SD)	33.7 ± 1.2
Age postnatal days, mean (range)	7 (4–19)
GA age at measurement, mean (range)	39 + 5 (38 + 4–40 + 6)
Duration of respiratory support (days), mean (range)	0.025 (0–0.2) ^1^
Intraventricular haemorrhage (n)	0
40 week SEF Latency, ms	**83.8 ± 11.6**

GA: gestational age, SD: standard deviation, ms: miliseconds. GA: gestational age, SD: standard deviation, ms: miliseconds. The values are given as mean values with standard deviation (SD) or range. 1 In the group of term infants, only one neonate had CPAP for 5 h.

The clinical characteristics of preterm infants and term infants are shown in Table 1, Table 2. Two infants in the very preterm group had intraventricular haemorrhage: one infant born at 26 + 2 weeks' gestation had unilateral grade II IVH, and one infant born at 28 + 6 weeks' gestation had grade III IVH on the right and grade II IVH on the left. No infant had grade IV IVH, post-haemorrhagic hydrocephalus, or required neurosurgical intervention.

### 3.2 SEF waveform characteristics

**Fig. 1:**
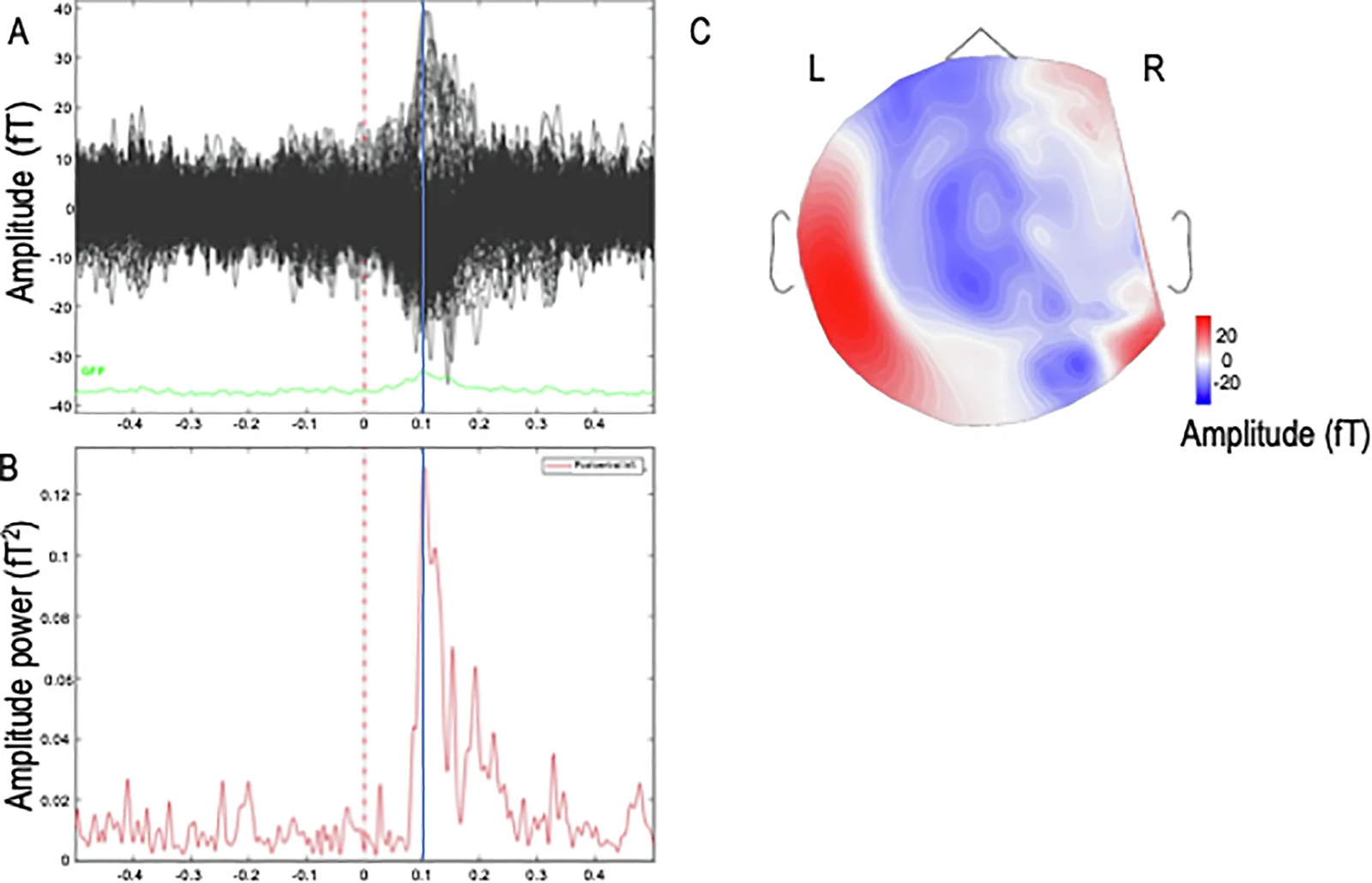
Representative SEF waveform in a preterm infant born at 29 + 5 weeks' GA. The dotted line marks the tactile stimulus, denoted as time point 0 ms. A. Butterfly plot, which displays all MEG channels superimposed. The y-axis represents the activation strength in femto Tesla (fT), GFP (global field power) provides a summary statistic for the overall activation strength over all channels. B. Plot of the activation strength over time within the left postcentral gyrus as estimated by source analysis. The latency to the first peak in this preterm infant was 108 ms. C. Magnetic field topography over the MEG sensor helmet at 108 ms (peak activation in panels A and B, marked as continuous line in A). Note that the infant's head was positioned to lean to the left to minimized the distance of the activated cortex to the ipsilateral MEG sensors.

Across all infants, somatosensory evoked field (SEF) recordings showed slow deflections consistent with previously described neonatal SEF morphology. Waveforms obtained in preterm infants at 36 weeks' GA demonstrated a similar overall morphology to those observed in term infants at 40 weeks' GA (Fig. 1).

### 3.3 SEF latencies in preterm vs. term infants

**Fig. 2:**
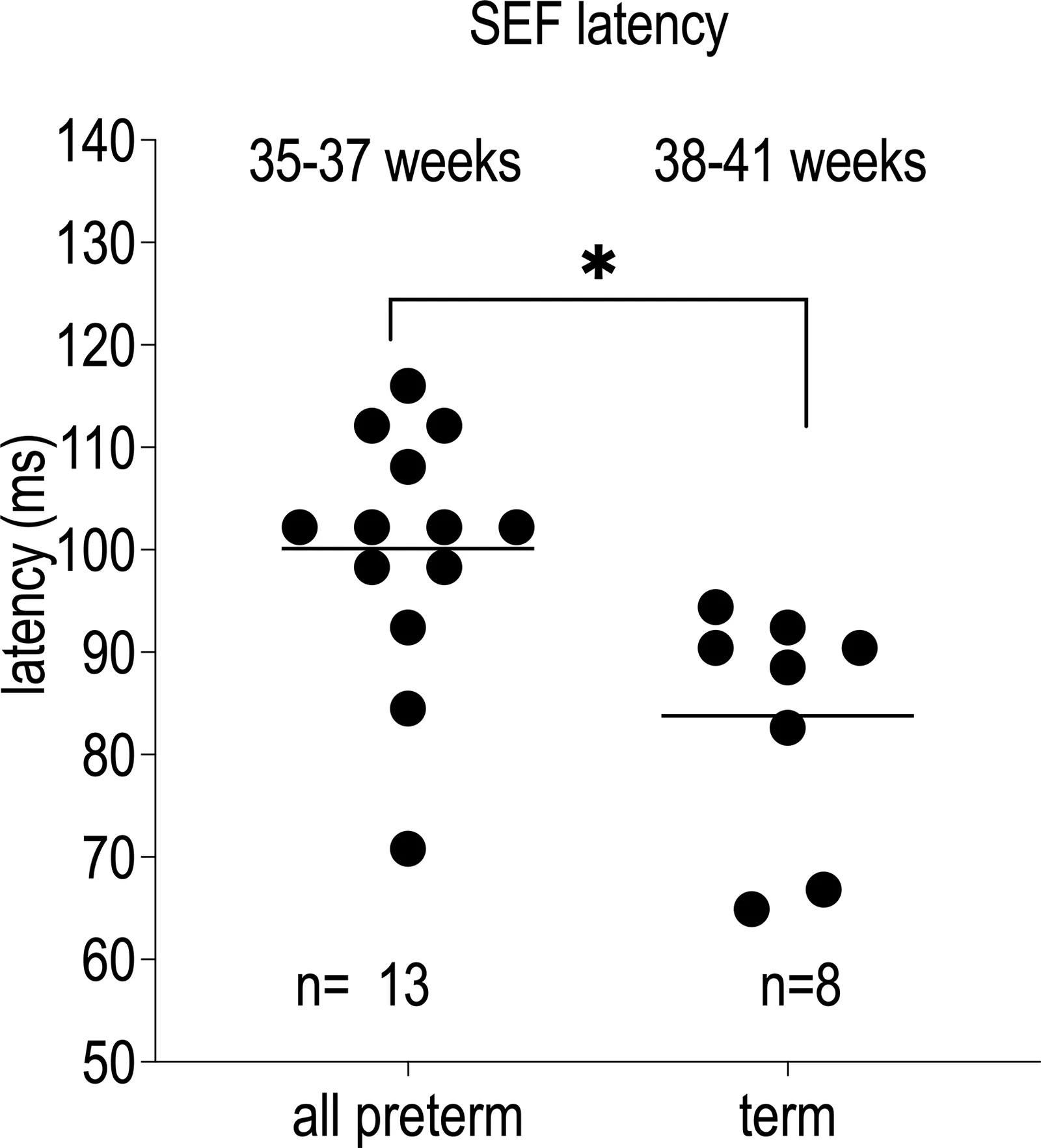
Shows the SEF latencies of all preterm infants (left column) versus term infants (right column). Values for SEF latencies are plotted on the y-axis. Each dot corresponds to a measurement of one infant. The horizontal line indicates the mean value. The measurement of preterm infants took place at a gestational age of 35–37 weeks of GA, term infants were measured at 38–41 weeks of gestational age. The difference between the latencies at preterm age and term age was significant (100.1 ± 12.2 ms vs. 83.8 ± 11.6 ms, *p* = 0,02 (one-way ANOVA including a correction for multiple comparisons).

Latencies to the first SEF deflection were longer in preterm infants at 36 weeks' GA compared to term infants at 40 weeks' GA (Fig. 2). Mean latency decreased by 16.3 ms from 36 to 40 weeks, corresponding to a reduction from 100.1 ± 12.2 ms at 36 weeks to 83.8 ± 11.6 ms at 40 weeks. The difference between the latencies at 36 weeks of GA and 40 weeks of GA was significant (*p* Value 0.02, one-way ANOVA and Dunn's multiple comparison post-hoc test).

**Fig. 3:**
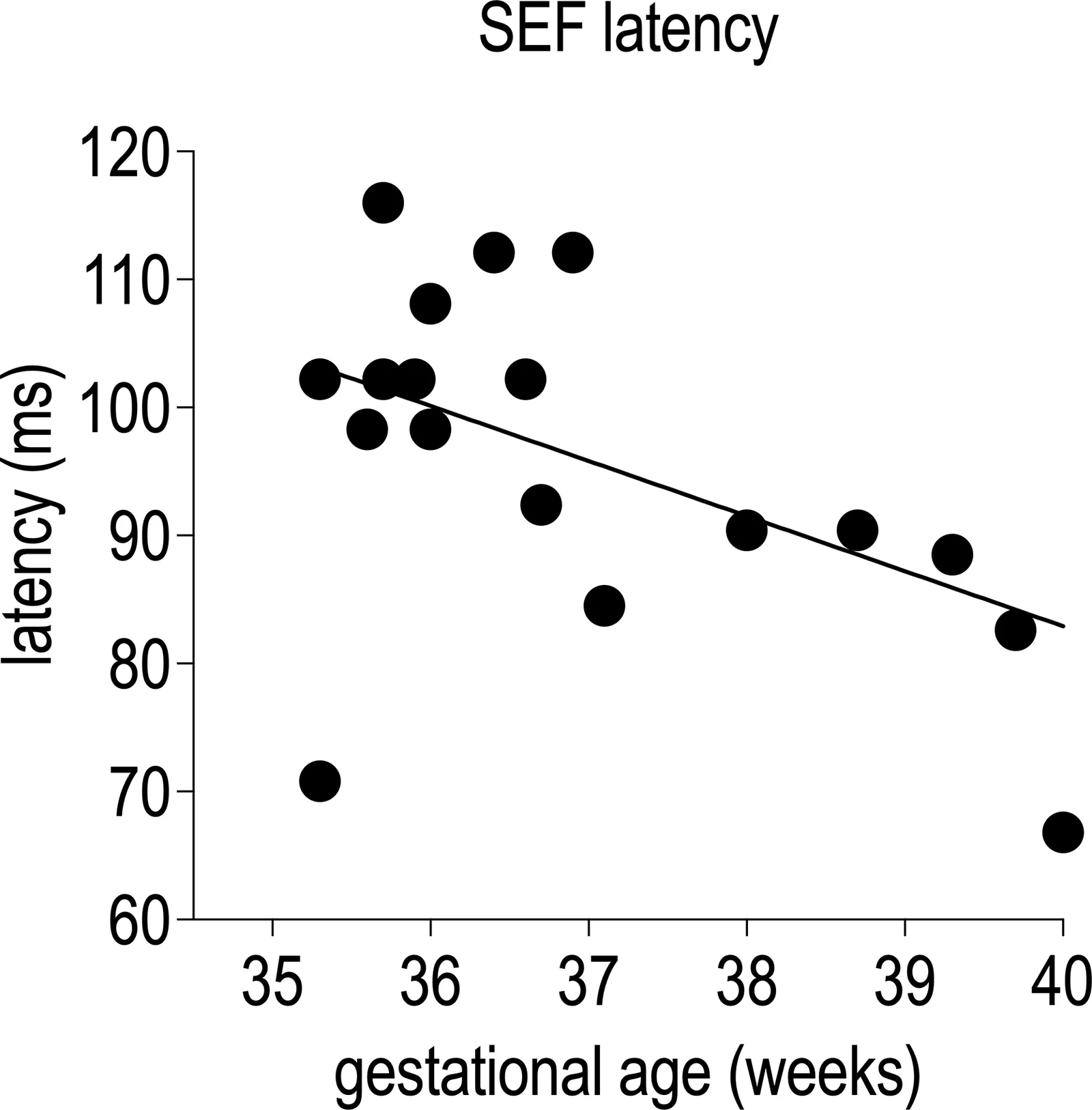
Linear relationship between SEF and gestational age at measurement.

A significant linear relationship between GA and SEF latency was observed: y = −4.3 × GA + 254.9 ms; R^2^ = 0.33 (Fig. 3).

### 3.4 SEF latencies in very preterm vs. moderate/late preterm infants

**Fig. 4:**
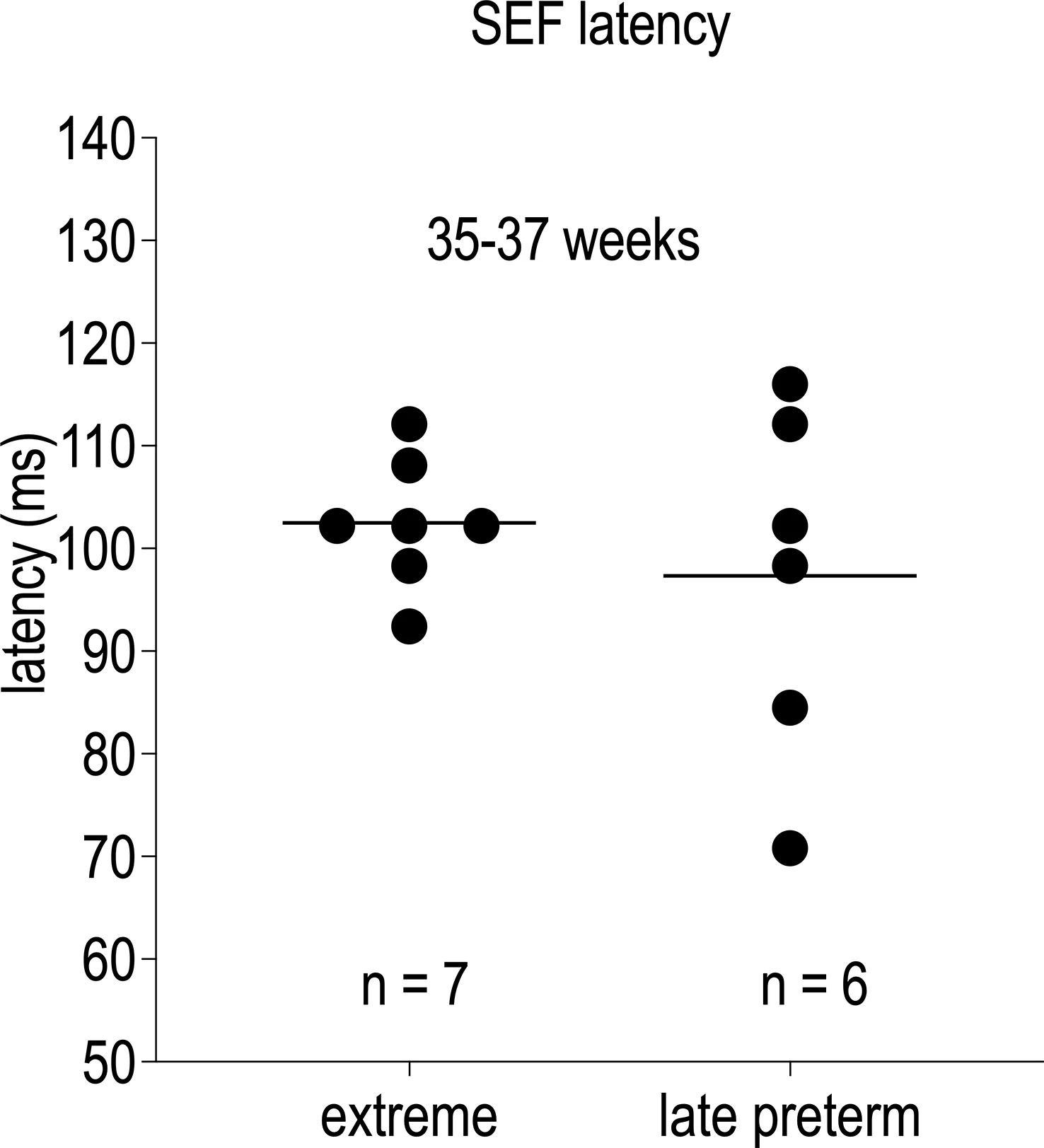
The group of preterm infants was divided in very/extreme preterm (left column) and late preterm neonates (right column), shown on the x-axis. Values for SEF latencies are plotted on the y-axis. Each dot corresponds to a measurement of one infant. The horizontal line indicates the mean value. The measurement of both groups took place at 35–37 weeks of GA. The difference between the groups was not significant. (*p* > 0.9, one-way ANOVA including a correction for multiple comparisons).

SEF latencies at 36 weeks' GA did not differ significantly between very preterm infants (26–31 weeks' GA) and moderate/late preterm infants (32–36 weeks' GA). Mean latencies were 102.5 ± 6.4 ms and 97.3 ± 17.1 ms, respectively (Fig. 4).

### 3.5 Neurodevelopmental follow-up of preterm infants

As part of our preterm follow-up programme, 5 of the 7 very preterm infants (26–32 weeks of gestational age) were assessed. 4 children completed the cognitive Scale of the BSID-III at a mean corrected age of 26.5 months (range 23–29, SD 2.6). Whereas 3 children had a composite cognitive Score in the normal range (90–110, mean 100, SD10), one infant had a poor result with a composite score of 55. The SEF latency of the latter infant did not differ to those with normal cognitive composite scores. In the case of another very premature baby, the two-year check-up has not yet taken place; the assessment using the Griffith scales at the age of 15 month showed a normal result. Two very preterm infants are lost to follow-up. We identified no cases of cerebral palsy. For moderate to late preterm infants, standardized follow-up was not available. One child from the latter group underwent developmental assessment with the BSID-III at 25 months and had a cognitive composite score of 80.

## 4 Discussion

### 4.1 Principal findings

In this pilot study, we report the feasibility of measuring somatosensory evoked fields (SEFs) in preterm infants at 36 weeks' gestational age using tactile stimulation with MEG. SEF waveforms were recordable in all infants and presented a neonatal morphology with slower and broader deflection than in adults. As expected, SEF latencies were longer at 36 weeks compared to 40 weeks' GA, and latencies decreased with advancing gestational age. No significant differences in SEF latency were observed between very preterm and moderate/late preterm infants. Follow-up assessments available for a subset of preterm infants revealed normal neurological development in our patient group at the age of 12–24 month.

### 4.2 Comparison with previous work

Previous studies have characterized SEF maturation in term infants and in preterm infants at 40 weeks' GA (Nevalainen et al., 2008; Nevalainen et al., 2012; Lauronen et al., 2006; Pihko et al., 2009). Our findings extend this work by demonstrating that SEF measurement is feasible earlier, i.e., at 36 weeks' GA, using tactile stimulation during natural sleep. Consistent with previous SEP and SEF studies (Bongers-Schokking et al., 1990; Trollmann et al., 2010; Tombini et al., 2009; Hrbek et al., 1973), preterm infants demonstrated prolonged latencies compared to term infants. The magnitude of latency reduction between 36 and 40 weeks (∼16 ms) was comparable to that reported in electrical SEP studies, although absolute latencies were longer in our study, likely due to methodological differences between tactile and electrical stimulation. Tactile stimulation activates mechanoreceptors and generates action potentials with slower transduction than simultaneous electrical activation of peripheral nerves, which may account for these differences (Caruso et al., 1994; Caruso et al., 1993).

### 4.3 SEF latencies, myelination and neurodevelopment

The progressive shortening of SEF latency from preterm to term age aligns with established concepts of increasing myelination in the peripheral and central nervous system(Gilmore et al., 1985; Lauronen et al., 2006; Pihko et al., 2009). Myelination facilitates rapid action potential conduction and is central to cortical network maturation. Disruptions in myelination are discussed as a pathway to neurodevelopmental impairment in preterm infants.

Interestingly, despite hypotheses that extrauterine development may slow or disrupt intrauterine myelination trajectories (Grotheer et al., 2023), we observed no latency differences between very preterm and moderate/late preterm infants at 36 weeks' GA.

The following explanations are possible: First of all, the somatosensory system may follow a robust developmental trajectory that is relatively resistant to moderate extrauterine influences.

Second, none of the very preterm infants had severe complications such as grade 4 IVH, hydrocephalus, or sepsis. Only one out of 4 very preterm infants in the study group had a severe cognitive delay at the age of 2 years. However, SEF latency was not prolonged in this child. So, the low-risk profile and the favourable neurodevelopmental outcome in the majority of the preterm infants might have resulted in similar latencies in very preterm and late preterm infants. Whether differences in SEF latency indicate cognitive impairment, needs to be investigated in a larger group of patients.

One methodological limitation of our study is that we measured the total conduction latency which consists of a peripheral and a central part. We did not record the peripheral nerve conduction time because it is technically challenging due to the small size of the nerves in premature babies and requires electrical stimulation of the median nerve. One might assume that peripheral latency differs greatly between premature and full-term babies and that this has a significant impact on the latency results. However, studies on peripheral nerve conduction velocity confirmed that there was only a difference of 3–4 m/s between the peripheral nerve conduction velocities of preterm infants at 36 weeks' gestation and those of term infants (Lori et al., 2018). In a SEP study conducted by our own research group (Trollmann et al., 2010), cervical latency was measured in preterm and term infants and then subtracted from the total latency to obtain the central conduction time. The central conduction time was significantly shortened by 15.6 ms between 36 weeks' and 40 weeks of GA which corresponds to the difference in our study, which was 16.3 ms. The latency from the wrist to the cervical region, by contrast, differed only slightly (11.0 vs 10.1 ms, mean values). These findings underline our hypothesis that extended latencies in preterm infants are mainly caused by a prolonged central pathway.

Because our study did not include MRI data, we were unable to determine the proportion of patients with white matter injury. Last but not least, SEPs only reflect the activity of the sensory nerve pathways via the spinothalamic tract to the cortex. The somatosensory system is one of the first to develop in utero from the 26th week of gestation on (Kostovic and Judas, 2010; Vanhatalo, s., and Lauronen, L., 2006) and is therefore possibly more conserved compared to, for example, frontal networks, which develop later in brain development. The absence of a latency difference in the somatosensory system does not rule out developmental changes in other functional systems that may be more directly relevant to later cognitive or executive outcomes. The absence of a latency difference in the somatosensory system does not rule out developmental changes in other functional systems that may be more directly relevant to later cognitive or executive outcomes. In this context, MEG studies investigating the oscillatory activity of the frontal lobe revealed evidence for an atypical frontal cortex development in preterm infants (Doesburg et al., 2011; Doesburg et al., 2013).

### 4.4 Limitations and future directions

Several methodological aspects of this pilot study should be considered when interpreting the findings. First, the sample size was small and derived from a single center, which limits statistical power and external validity. The lack of differences between very preterm and moderate/late preterm infants may therefore reflect limited power rather than true equivalence. At the same time, neonatal MEG is available at only a few specialized university centers in Germany, and the acquisition of high-quality recordings in preterm infants is technically demanding. Moreover, only clinically stable preterm infants without major complications such as severe IVH, sepsis, or hydrocephalus were eligible for measurement, restricting applicability to more vulnerable populations at an early age of 36 weeks of GA.

Second, SEF recordings were based on pneumatic tactile stimulation, which – although more infant-friendly than electrical stimulation - is less standardized across laboratories. Variability in stimulus force and transduction dynamics may contribute to the longer absolute latencies observed compared with electrical SEP studies, and may limit inter-laboratory comparability.

Third, each infant underwent only a single MEG recording. As a result, individual maturation trajectories could not be assessed, and age-related changes must be interpreted at the group level. Longitudinal studies in preterm infants are inherently challenging because of clinical instability at younger ages and the need to avoid sedation in older infants. A longitudinal design with repeated measurements in the same preterm infants at 36 and 40 weeks' GA would have provided a stronger comparison with term infants. In the present pilot study, this was not feasible because many clinically stable preterm infants were discharged before 40 weeks' GA, and return visits for MEG recordings would have been a burden for the families.

Fourth, the study lacked MRI data, preventing direct correlation of SEF latencies with white matter injury, tract integrity, or myelination markers. Mild or diffuse white matter abnormalities may therefore have remained undetected.

Finally, developmental follow-up was incomplete and did not extend into school age, when cognitive, attentional, and executive-function difficulties typically become apparent.

Further studies demand longitudinal measurements, MRI correlation, the assessment of higher-order responses and a longer follow-up until school age.

## 5 Conclusion

Our study findings provide an important foundation for future MEG studies of premature infants in early life. The integration of MEG with advanced neuroimaging is a promising approach for obtaining functional data on normal and abnormal brain development and maturation in premature infants.

## Acknowledgement and funding

The first author S.S. received a scholarship for a research leave from clinical work from Friedrich-Alexander-University. Additionally, the study was financially supported by the Johannes-and Frieda Marohn Foundation.

We found a linear relationship between gestational age at measurement and the SEF latencies. Latencies shortened with increasing gestational age. The relationship was significant. y = −4.3 × GA + 254.9 ms; R^2^ = 0.33.

## CRediT authorship contribution statement

**Stephanie C. Schüssler:** Writing – original draft, Investigation, Visualization. **Fabian B. Fahlbusch:** Writing – review & editing. **Undine Niederreiter:** Investigation, Data curation. **Martin Kaltenhäuser:** Investigation, Data curation. **Heiko Reutter:** Resources. **Regina Trollmann:** Conceptualization. **Stefan Rampp:** Writing – review & editing, Supervision.

## Declaration of competing interest

None of the authors have potential conflicts of interest to be disclosed.

## Figures and Tables

**Supplementary Fig. 1:**
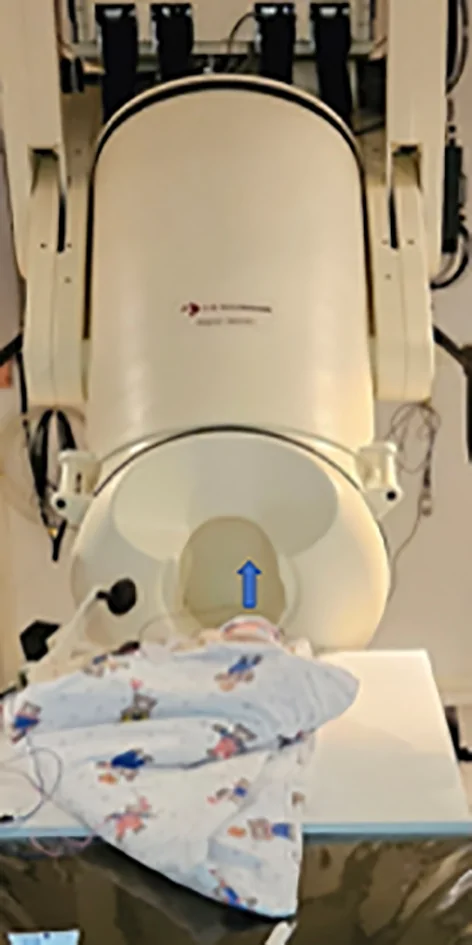
Photograph of the experimental setup for the SEF measurement.
